# Intraventricular Undifferentiated Pleomorphic Sarcoma: A Case Report

**DOI:** 10.7759/cureus.876

**Published:** 2016-11-14

**Authors:** Emily P Sieg, Hayk Stepanyan, Russell Payne, Elizabeth Frauenhoffer, Charles S Specht, Sara Langan, Elias Rizk

**Affiliations:** 1 Department of Neurosurgery, Penn State Hershey Medical Center; 2 Medical Student, Penn State College of Medicine; 3 Pathology, Penn State Hershey Medical Center

**Keywords:** cranial spindle cell tumor, intraventricular mass, spindle cell tumor, undifferentiated pleomorphic sarcoma

## Abstract

Undifferentiated pleomorphic sarcoma is a histologic diagnosis based on cell morphology. These tumors are found throughout the body. They are rarely found in the central nervous system and almost never occur as a primary intraventricular tumor. We present the unusual case of a 68-year-old woman with an intraventricular undifferentiated pleomorphic sarcoma. We go on to discuss the clinical presentation, radiographic characteristics, and management paradigm for these rare lesions. Our patient presented with acute confusion, inability to balance a checkbook, and gait imbalance. CT and MRI demonstrated a 4 x 3.6 x 3.6 cm enhancing lesion in the left lateral ventricle abutting the foramen of Monro. Pathology revealed an undifferentiated pleomorphic sarcoma.

## Introduction

Undifferentiated pleomorphic sarcoma (UPS) is a synonym for the more commonly reported malignant fibrous histiocytoma (MFH) as per the 2002 World Health Organization classification [1]. These are commonly found in the extremities and in the retroperitoneum [2-3]. Intracranial undifferentiated pleomorphic sarcoma has rarely been reported. The first report of the occurrence of UPS in the central nervous system was in 1976 by Gonzalez-Vitale et al [4]. UPS can metastasize to the brain or arise from the central nervous system. Primary central nervous system undifferentiated pleomorphic sarcoma can be divided into three types: meningeal UPS arising from undifferentiated multipotential cells found in the meninges, parenchymal UPS arising from perivascular mesenchymal cells or the pial sheath surrounding Virchow-Robin spaces, and ventricular UPS arising from mesenchymal precursor cells of the tela choroidea [5]. There have only been two reported cases of intraventricular UPS: one by Berchtenbreiter et al., in 1996 and one Baehring et al., in 2001 [6-7]. Thus, we present here the rare case of a primary undifferentiated pleomorphic sarcoma that arose within a cerebral ventricle.

## Case presentation

**Figure 1:**
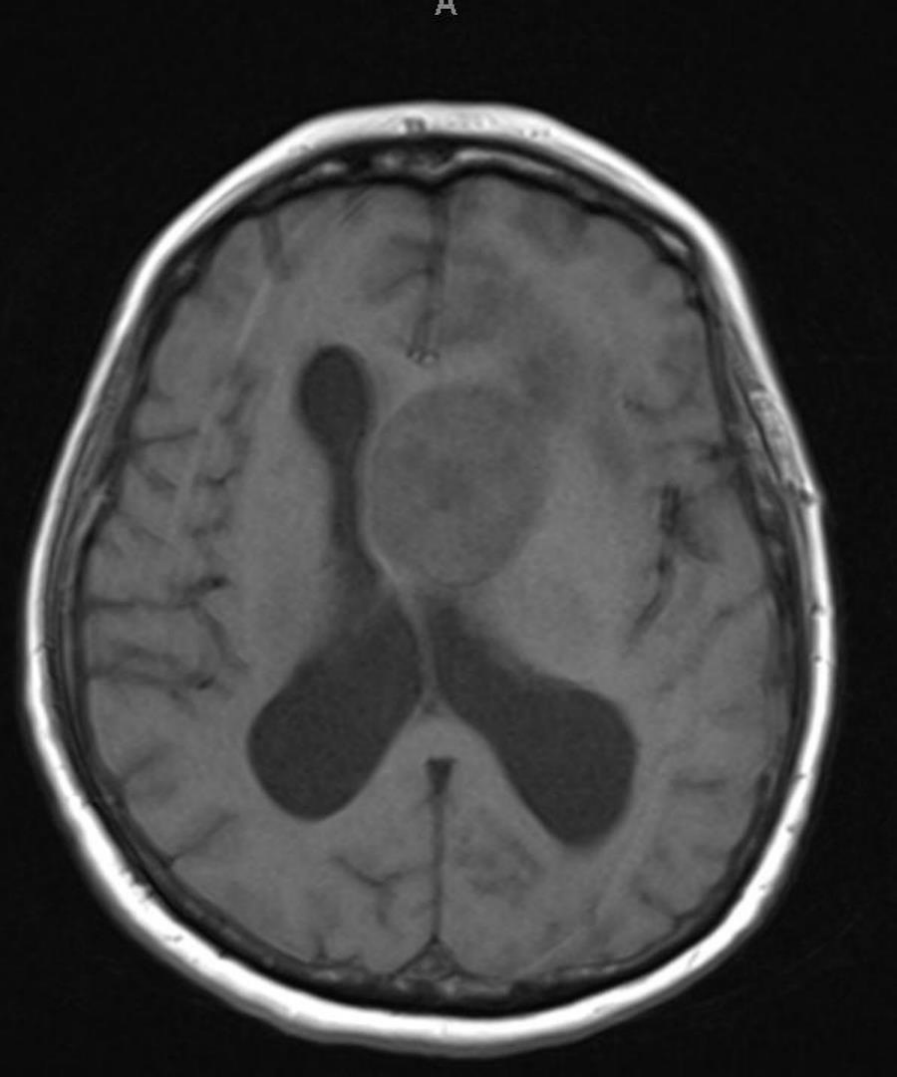
Initial MRI (pre-contrast) T1 axial pre-contrast MRI showing a hypodense lesion in the left lateral ventricle.

**Figure 2:**
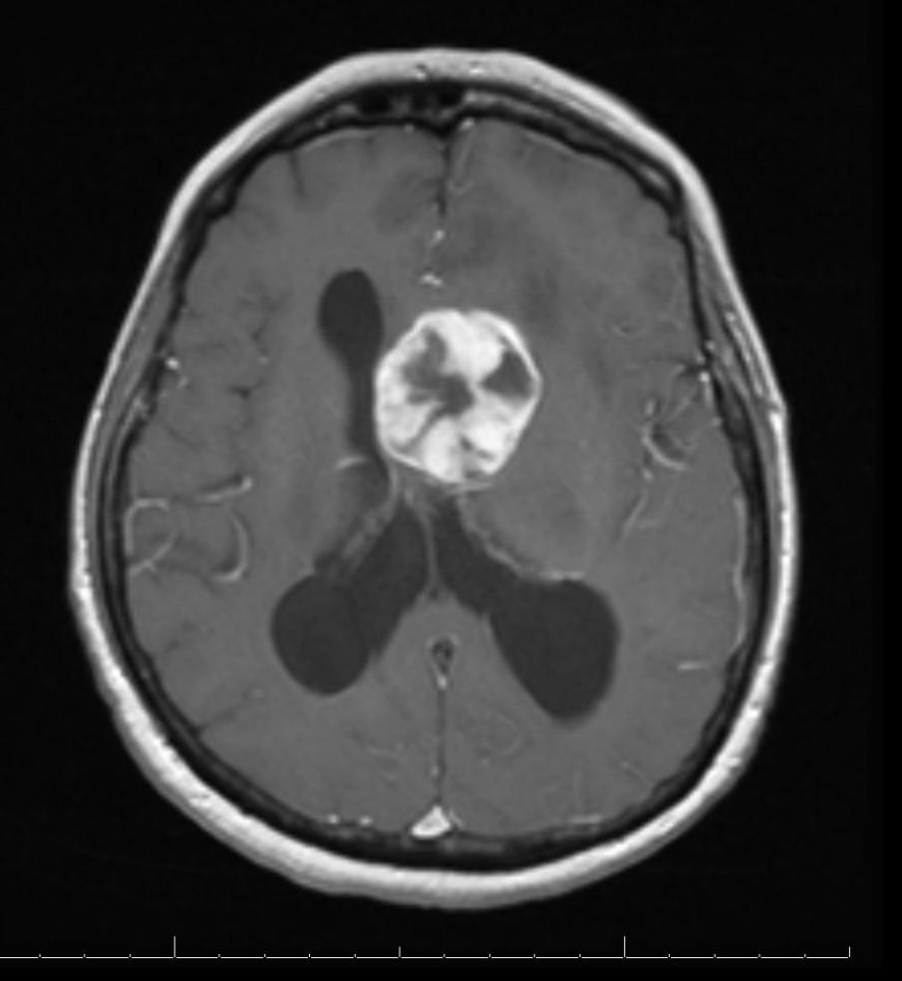
Initial MRI (post-contrast) T1 axial post-contrast MRI showing the heterogeneous enhancement of the lesion.

**Figure 3:**
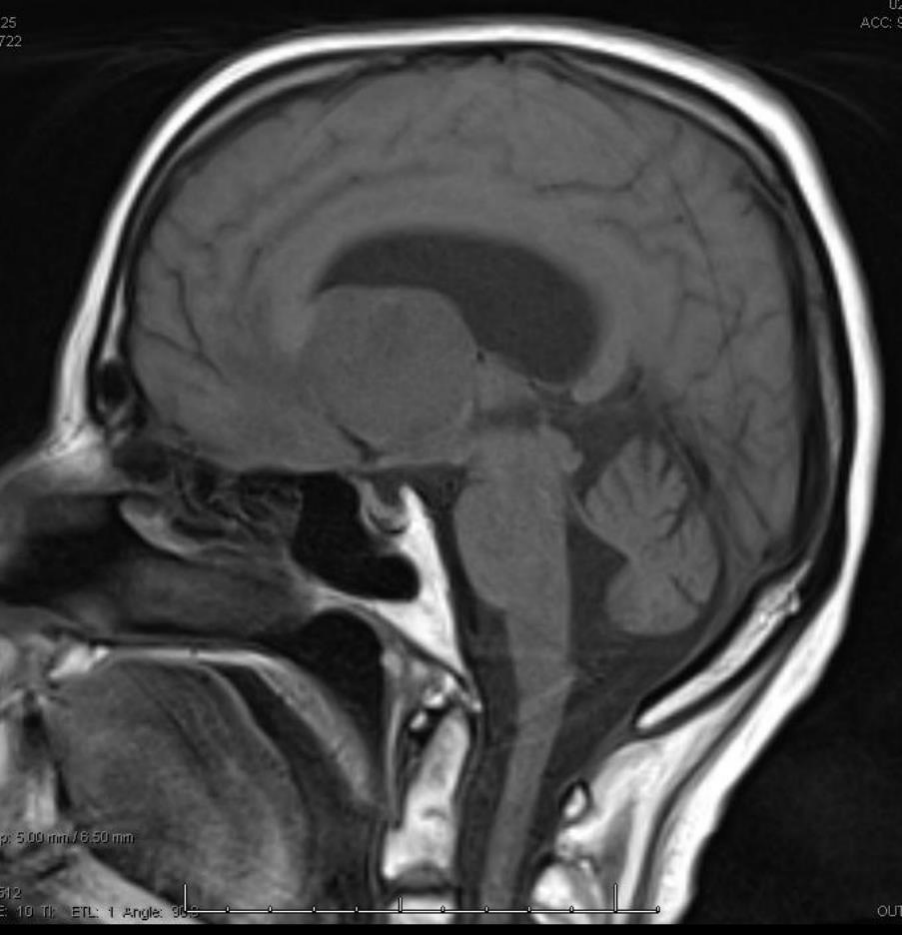
Initial MRI (pre-contrast) T1 sagittal pre-contrast MRI scan showing mass effect of the lesion on the foramen of Monro.

**Figure 4:**
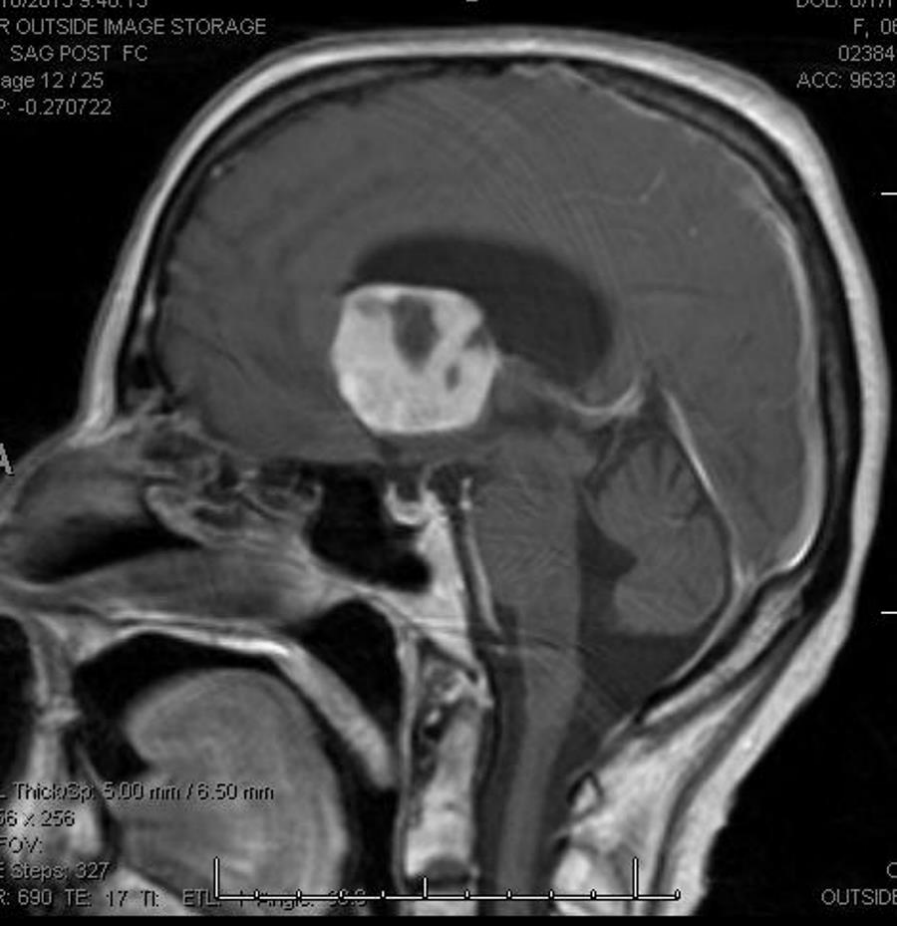
Initial MRI (post-contrast) T1 sagittal post-contrast MRI scan showing mass effect of the lesion on the foramen of Monro.

**Figure 5:**
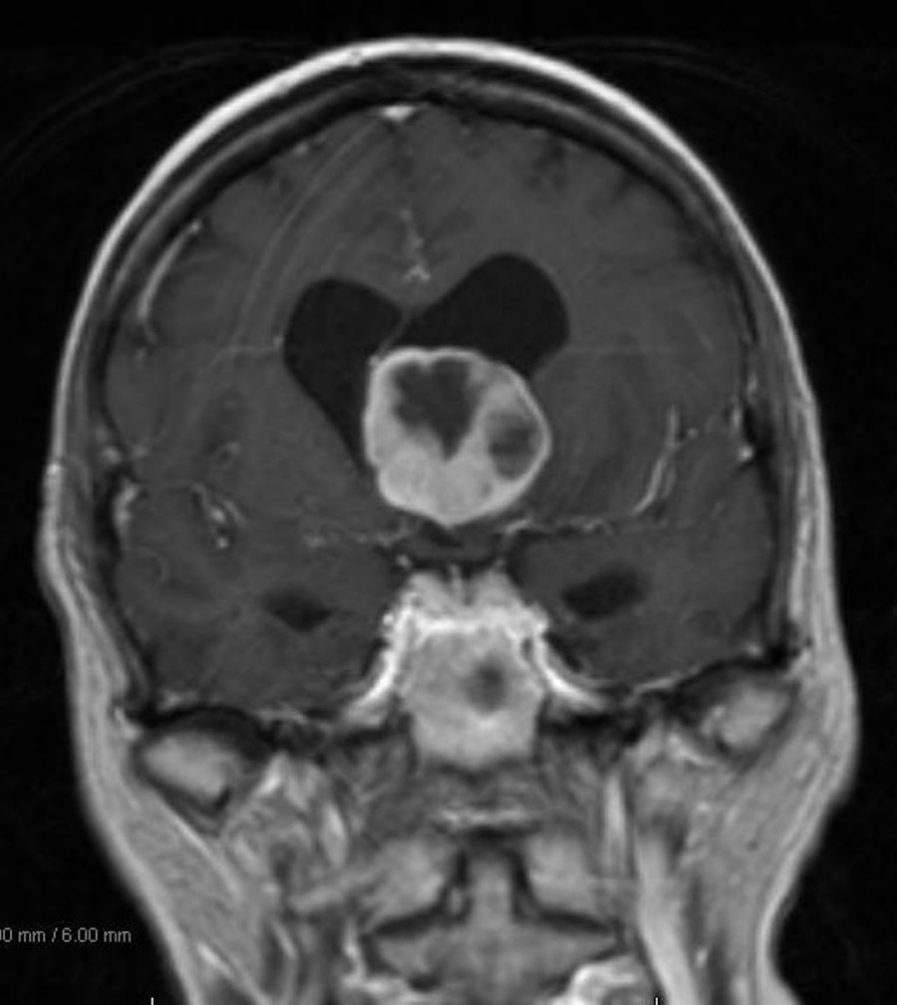
Initial MRI (post-contrast) Coronal post-contrast MRI demonstrating the mass effect caused by the heterogeneously enhancing lesion.

Our patient is a 68-year-old, right-hand female with a history of breast cancer status post-lumpectomy followed by radiation in 1993, (further details regarding previous her breast cancer are not available). She presented to an outside institution for acute confusion, inability to balance her checkbook, and slowly progressing gait imbalance with left leg “dragging.” She was evaluated initially with a head CT scan that demonstrated a heterogeneous isodense intraventricular mass lesion predominantly in the left lateral ventricle with surrounding left frontal lobe edema and hydrocephalus. She subsequently had an MRI of the brain, which demonstrated a heterogeneously enhancing T2 hyperintense, T1 hypointense mass in the left lateral ventricle near the foramen of Monro (Figure 1, Figure 2, Figure 3, Figure 4, Figure 5). This lesion measured 4 x 3.6 x 3.6 cm and was associated with moderate hydrocephalus due to mass effect on the foramen of Monro.

Following the MRI, she was referred to Penn State Hershey Medical Center. The patient agreed to participate and was explained the nature and objectives of this study, and informed consent was formally obtained. No reference to the patient's identity was made at any stage during data analysis or in the report.

Given her history of breast cancer and concern for metastatic spread, additional radiographic studies--including CT examination of the chest, abdomen, and pelvis--were obtained and did not reveal any other lesions. Of interest, the patient had a previous MRI of the brain in 2013 that showed no mass lesion or ventriculomegaly. Her presenting review of systems was positive for urinary incontinence, gait dysfunction, and difficulty with higher-order tasks; however, the patient reported no headaches, seizures, or visual complaints. On physical examination, she was oriented to person only, but the remainder of her examination was non-focal.

She underwent a left-sided interhemispheric transcallosal resection of the intraventricular tumor as well as placement of an external ventricular drain (EVD) which remained clamped at the end of the case. The patient had a seizure which broke with Ativan in the post-anesthesia care unit, and she was loaded with Keppra. She was admitted to the neuro-intensive care unit for close observation. Her intracranial pressure remained less than 10 mmHg, and her neurologic examination remained stable thus her EVD was removed 48 hours postoperatively. She was seen by our Neuro-Oncologist, and chemotherapy and radiation were discussed. The patient’s daughter elected to undergo further treatment and oncologic care closer to home. She worked with physical therapy, occupational therapy, and speech therapy and was discharged to rehab on postoperative day five.

**Figure 6:**
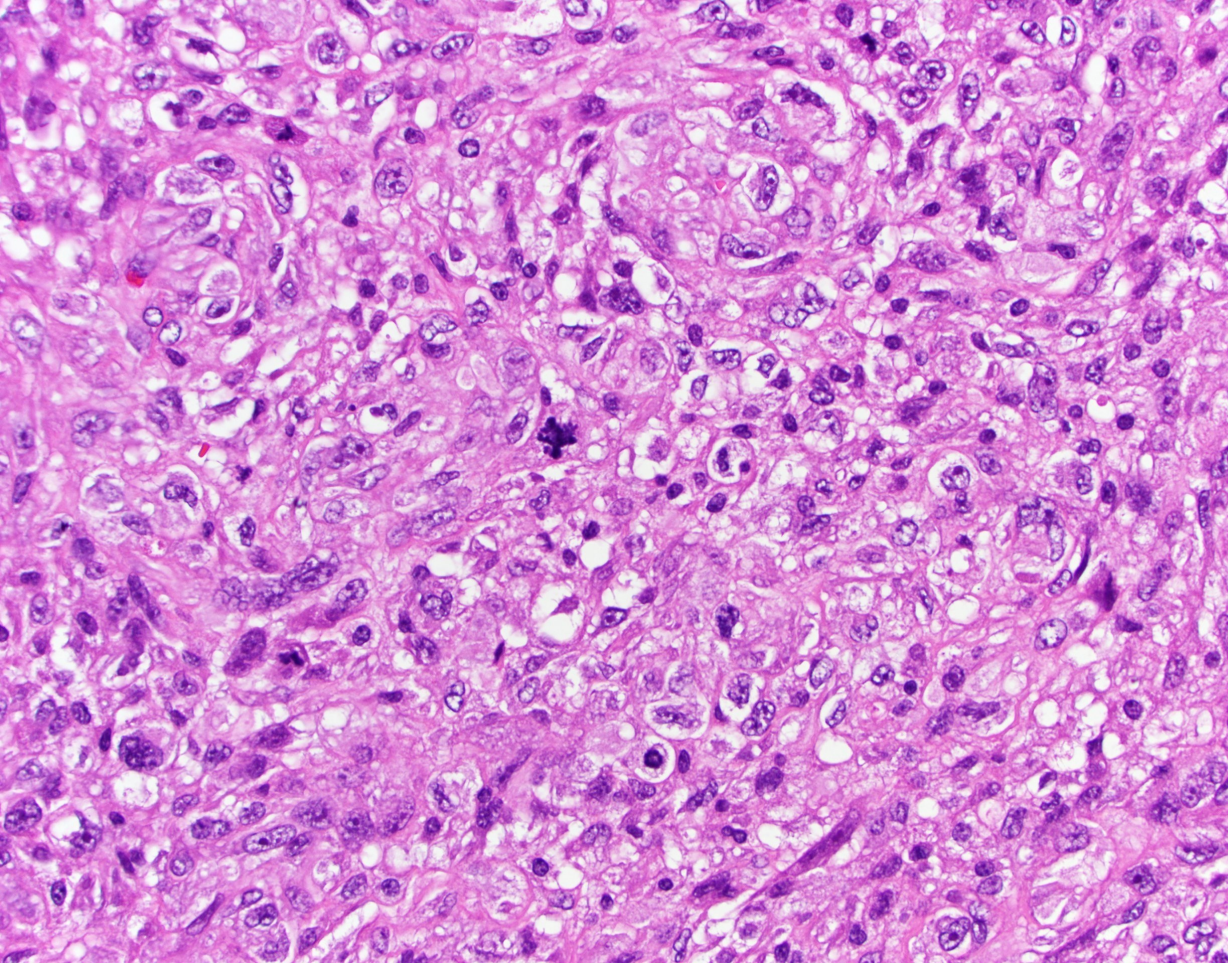
H&E, 400x The tumor is composed of spindle cells with pleomorphic nuclei. The cells form a sweeping storiform (straw mat-like) pattern. An atypical mitosis is present.

**Figure 7:**
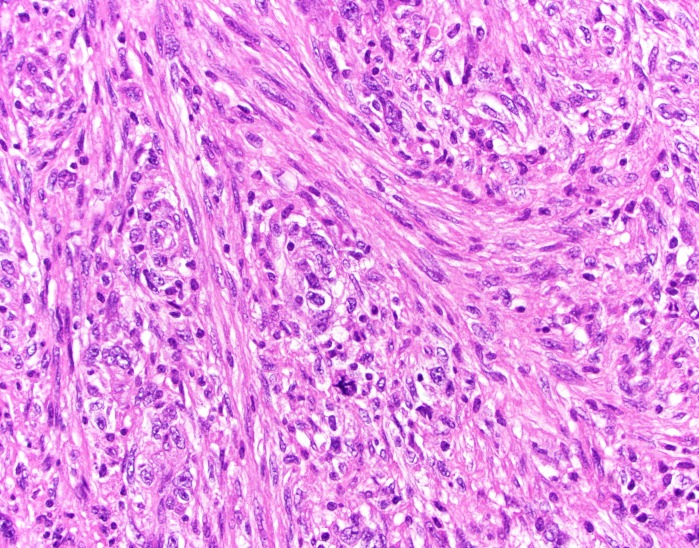
H&E, 400x The tumor is composed of spindle cells with pleomorphic nuclei. The cells form a sweeping storiform (straw mat-like) pattern. An atypical mitosis is present.

**Figure 8:**
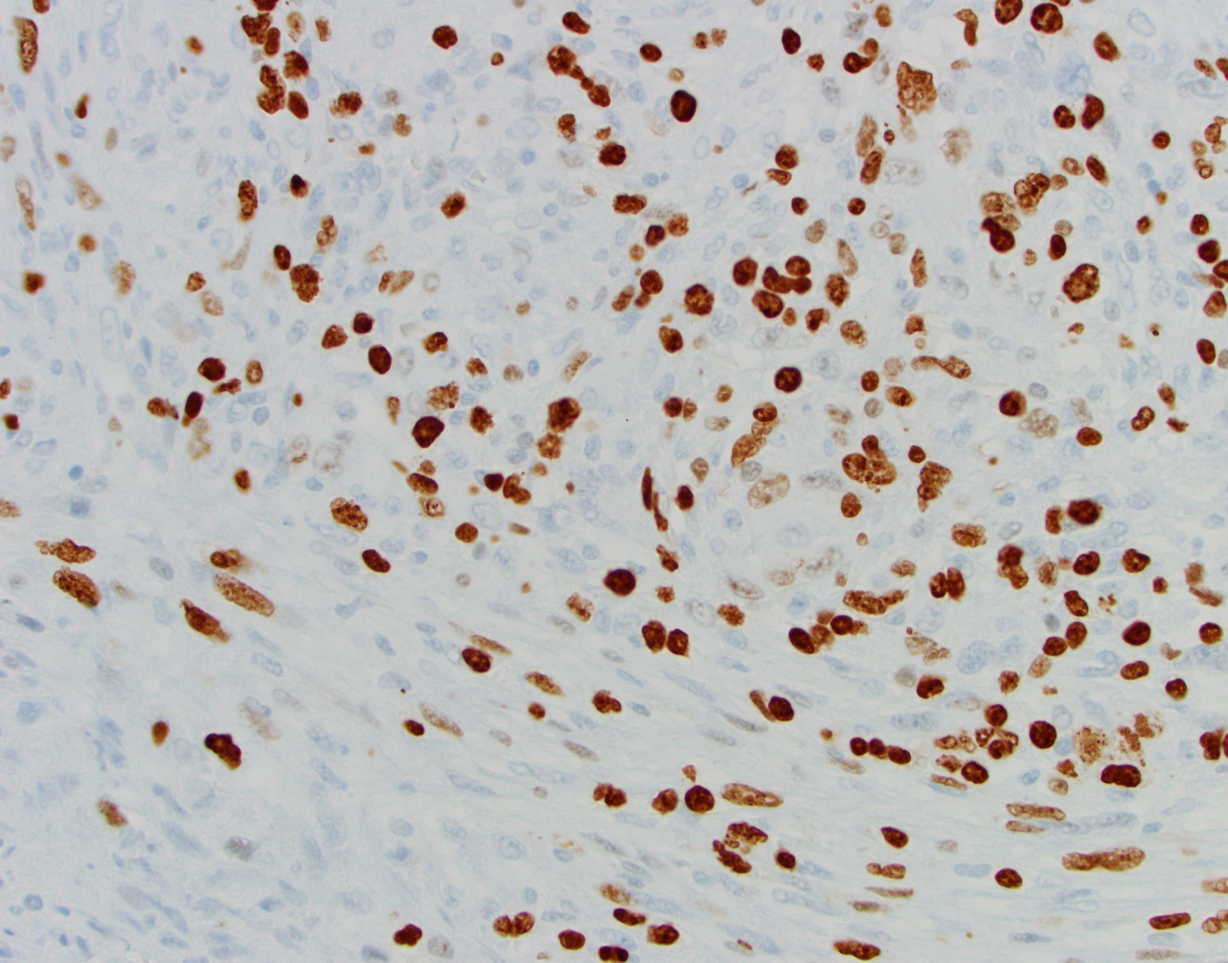
Ki-67, 400x Brown nuclear staining for this proliferation marker is seen in many tumor cells.

Pathology revealed undifferentiated pleomorphic sarcoma (Figure 6, Figure 7, Figure 8).

This high-grade spindle neoplasm was composed of cells with marked nuclear pleomorphism. The tumor cells had a storiform growth pattern. Immunohistochemical markers for glioma (glial fibrillary acidic protein [GFAP]; S100), meningioma (epithelial membrane antigen [EMA]), solitary fibrous tumor (CD34), hemangiopericytoma (CD34; BCL-2), angiosarcoma (CD31; CD34), muscle tumors (desmin; smooth muscle actin [SMA]), carcinoma (cytokeratin CAM 5.2; EMA), and melanoma (HMB45; MART1/MelanA; S100) were negative in the neoplastic cells. The tumor had significant necrosis. The mitotic index was 20 mitoses per 10 high-power fields (viewed at 400 xs), and the Ki-67 nuclear labeling index was 35%.

In summary, we present a 68-year-old patient who presented with an intraventricular mass which upon resection was morphologically and immunohistochemically consistent with undifferentiated pleomorphic sarcoma. This neoplasm is a diagnosis of exclusion; it may be diagnosed when the histology is that of a malignant spindle cell neoplasm, and evaluation with immunohistochemistry does not reveal findings diagnostic for other sarcomas, carcinomas, or malignant melanoma.

## Discussion

The common differential diagnosis for intraventricular lesions in adults includes choroid plexus papilloma or carcinoma, ependymoma, central neurocytoma, subependymal giant cell astrocytoma, lymphoma, meningioma, and glioblastoma. Hamlat et al., presented 49 reported intracranial pleomorphic sarcomas [8]. They found that UPS may occur at any age and with no sex predominance. Their average patient age was 38 years--although 26% of cases occur among children. The majority of the tumors were supratentorial, with the most frequent sites being in the frontal and temporal lobes. Intraventricular undifferentiated pleomorphic sarcoma is an extremely rare lesion reported only twice in the literature to date.

Surgery alone results in poor survival rates and poor local control. It would, therefore, seem logical that adjuvant radiotherapy would demonstrate a survival benefit; however, the optimal dose has not yet been proven. Even with aggressive chemotherapy and radiation, median survival was only 27 months due to local progression with occasional metastasis and cerebrospinal fluid dissemination according to Hamlat et al.’s series [8]. Doxorubicin and ifosfamide have been used in the treatment of intracranial UPS due to their activity against soft tissue sarcomas as reported by Akimoto et al [9]. Ham et al., reviewed the use of a high-dose methotrexate-based regimen in 17 cases of bone UPS, and they found that it dropped the recurrence/metastasis rate from occurring at an average of 17 months to no recurrence during an average follow-up period of 10 years. Although primary bone UPS is highly responsive to this protocol combined with resection, there has been no report discussing the usefulness in primary intracranial UPS [10]. Unfortunately, treatment modality has not impacted overall survival rates which remain poor, between 7.2 and 27 months.

## Conclusions

In conclusion, undifferentiated pleomorphic sarcomas are uncommon, and those arising from the cerebral ventricles are especially rare. Here, we present a case of primary undifferentiated pleomorphic sarcoma of the cerebral ventricle in a 68-year-old woman. This tumor type should be differentiated from choroid plexus papilloma or carcinoma, ependymoma, central neurocytoma, subependymal giant cell astrocytoma, lymphoma, meningioma, and glioblastoma. Extensive tumor sampling, careful microscopic examination, and immunohistochemical staining may aid in a diagnosis of intraventricular primary undifferentiated pleomorphic sarcoma. This case highlights the need for further investigation of these rare-but-aggressive and deadly intraventricular lesions and stresses the importance of further research in this area. Further studies are required to investigate the optimal radiation dose and the use of chemotherapeutic regimens such as high-dose methotrexate.
